# Role of Psychiatric Intensive Care Units in Preventing Long Term Admissions in Psychiatric Hospitals

**DOI:** 10.1192/bjo.2025.10224

**Published:** 2025-06-20

**Authors:** Aqsa Shahbaz, Noman Ahmed, Farasat Ali, Nimra Mir, Faiqa Jannat

**Affiliations:** Punjab Institute of Mental Health Lahore, Lahore, Pakistan

## Abstract

**Aims:** This study aims to assess the role of psychiatric intensive care units (PICU) in preventing long-term admissions in psychiatric facilities which is a major issue in developing countries like Pakistan.

**Methods:** It was a retrospective cohort study. Data obtained from patients’ admission and discharge registers from Psychiatric ICU and two inpatient units in Punjab Institute of Mental Health Lahore for a time period of 6 months from November 2023 till April 2024 was studied and length of stay in PICU was compared with other units.

**Results:** 82% of PICU patients (n=110) were discharged within 6 days(S.D±3.08) after stabilization, with follow-up in OPD, while the remaining 18% were transferred to inpatient Unit B (n=52) for further management with average stay of 12 days.(S.D±5.23) Only 53% of the patients (n=26) admitted in Unit D (operating without PICU) were discharged, with the rest remaining hospitalized. Unit D had a longer average hospital stay of 41 days. The units were similar in demographic features but varied in treatment programmes and involvement of family in treatment.

**Conclusion:** The results of the study are promising in favour of PICU as it succeeded in reducing length of stay in the hospital and challenges the social perception of psychiatric facilities as a place of incarceration. Rapid mobilization of resources and active involvement of family during the management were important factors impacting the length of stay. There is further room for research for role of PICU in psychiatry wards in multidisciplinary hospitals.

